# Research on the Mechanism of Hypoxia Tolerance of a Hybrid Fish Using Transcriptomics and Metabolomics

**DOI:** 10.3390/biology14101462

**Published:** 2025-10-21

**Authors:** Yuhua Tang, Jiayi Yang, Chunchun Zhu, Hong Zhang, Li Hu, Wenting Rao, Xinxin Yu, Ming Wen, Min Tao, Shaojun Liu

**Affiliations:** 1 Engineering Research Center of Polyploid Fish Reproduction and Breeding of the State Education Ministry, College of Life Sciences, Hunan Normal University, Changsha 410081, China; 19310032129@163.com (Y.T.); 15067848138@163.com (J.Y.); 19307411565@163.com (C.Z.); zhanghong98@hunnu.edu.cn (H.Z.); 17872348328@163.com (L.H.); rdmnei@outlook.com (W.R.); yxx1758155428@163.com (X.Y.); minmindiu@126.com (M.T.); 2 Yuelushan Laboratory, Changsha 410128, China

**Keywords:** hybrid fish, transcriptomics, metabolomics, hypoxia tolerance, gills

## Abstract

**Simple Summary:**

A hybrid fish, called BTB, created by crossing two different species, showed a significantly improved ability for adapting in low-oxygen water compared to its parents, with a 20% increase in tolerance. However, the mechanism of hypoxia tolerance in BTB remained unclear. In this study, we found that gills physically adapted to hypoxia in BTB. By analyzing changes in genes and metabolites, potential biological pathways involved in stress response were identified. This study pinpointed 12 key genes that were likely responsible for responding to a hypoxic environment. Furthermore, we discovered that changes in lipid and amino acid metabolism (specifically in glycerophospholipid, ether lipid, arachidonic acid, and arginine/proline pathways) are involved in hypoxic response in fish. In short, this research identified the genetic and metabolic basis for hypoxia tolerance of hybrid fish, providing a foundation for future studies.

**Abstract:**

The novel hybrid fish BTB, derived from crossing blunt snout bream (*Megalobrama amblycephala*, BSB) and topmouth culter (*Culter alburnus*, TC), exhibits markedly hypoxia tolerance in aquaculture. In this study, hypoxic treatment experiments confirmed that, comparing to its original parent BSB, the tolerance to low oxygen of BTB increased by 20.0%. Furthermore, a comparative analysis of the transcriptome and metabolome was performed using gill tissues from BTB exposed to normoxic and hypoxic conditions. Under hypoxic conditions, BTB displayed adaptive modifications in gill lamellae and hemocytes. Transcriptomic profiling identified 789 differentially expressed genes (DEGs), with 298 upregulated and 491 downregulated, enriched in pathways including apoptosis, NK cell-mediated cytotoxicity, MAPK/TNF/Toll-like receptor signaling, and HIF-1/FoXO signaling pathways. Twelve hypoxia-related candidate genes (*egln3*, *im_7150988*, *znf395a*, *hif-1an*, *mknk2b*, *pck2*, *ero1a*, *igfbp-1a*, *vhl*, *bpifcl*, *egln1a*, and *ccna1*) were screened and validated as potential contributors to hypoxia tolerance. Metabolomics analysis revealed a total of 108 differential metabolites (78 upregulated and 30 downregulated), predominantly linked to Arginine and proline metabolism, Ether lipid metabolism, Arachidonic acid metabolism, and Glycerophospholipid metabolism. Association analysis of transcriptomics and metabolomics revealed that the DEGs and DMs were enriched in the pathways of glycerophospholipid metabolism, ether lipid metabolism, arachidonic acid metabolism, and arginine and proline metabolism. In summary, BTB exhibited relatively high hypoxia tolerance, and 12 candidate genes related to hypoxia tolerance were identified. These findings laid a foundation for further investigation into the mechanisms of hypoxia tolerance improvement in hybrid fish.

## 1. Introduction

Dissolved oxygen (DO) is an essential factor for fish growth in aquaculture. The DO level in water is influenced by various factors [1]. Commercial high-density aquaculture, pollution, and environmental factors such as temperature, ammonia–nitrogen concentration, and salinity can affect DO levels in water bodies [2,3]. Most fish experience a floating head phenomenon, leading to suffocation and death, when DO levels drop below 1.0 mg/L [4]. Hypoxic stress poses a major challenge to fish survival in aquaculture. In recent decades, the extent, frequency, and severity of hypoxia among aquatic animals have increased globally [5]. Hypoxia can cause significant economic losses to the aquaculture industry. Reasonably, research on the mechanisms of hypoxia adaptation in fish is helpful for improving the economic outcomes of aquaculture. Additionally, establishing hypoxia-tolerant fish strains through breeding techniques can provide high-quality germplasm resources for the aquaculture industry.

When faced with hypoxic stress, fish have certain defensive measures to adapt to the hypoxic environment. Adaptation to hypoxia is facilitated by coordinated changes, including behavioral reductions in swimming and feeding, morphological and physiological adjustments in the gills, increased erythrocyte numbers to enhance oxygen affinity, and a metabolic shift toward anaerobic glycolysis [6,7,8]. For example, goldfish (*Carassius auratus*) increase their respiratory surface area by expanding the gill lamellae to enhance contact with DO in water bodies under hypoxic conditions [9]. Superoxide dismutase (SOD) and Catalase (CAT) act in the gills of the common carp (*Cyprinus carpio*) to scavenge reactive oxygen radicals, thereby reducing oxidative stress [10,11]. In Nile tilapia (*Oreochromis niloticus*), hepatic glycogen utilization increases when they experience acute hypoxia [12]. Hypoxic stress in naked carp (*Gymnocypris przewalskii*) triggers an upregulation of Toll-like receptors (TLRs), a potential mechanism to defend against oxidative stress-induced tissue damage and physiological imbalances [13].

BTB, a hybrid bream, was generated in our laboratory by hybridizing female blunt snout bream (*Megalobrama amblycephala*, BSB) with male topmouth culter (*Culter alburnus*, TC) and subsequently backcrossed with the male parent BSB [14,15]. BTB like other progenies generated from distant hybridization exhibits rapid growth, flavorful meat, and strong stress resistance [16]. BTB was represented as a new variety of freshwater fish in China, and it exhibited hypoxia tolerance during aquaculture practices. However, the mechanisms of hypoxia tolerance in BTB remain unclear.

Transcriptomics and metabolomics are being used to explore new biological processes [17,18]. Transcriptomics enables the global analysis of gene expression, providing insight into functional gene expression under different conditions. This approach has been widely used to elucidate the molecular mechanisms of hypoxia adaptation in fish [19]. For example, the transcriptome results of the gill tissue of northern whiting (*Sillago sihama*) under hypoxic stress revealed changes in the expression patterns of 247 genes, which were primarily involved in immune response, energy metabolism, and oxidative defense [20]. Similar findings have been reported in other fish species such as large yellow croaker (*Larimichthys crocea*) [1], Nile tilapia [21], and Japanese pufferfish (*Takifugu rubripes*) [22]. Metabolomic analysis of gynogenetic mrigal carp (*Cirrhinus mrigala*) under cold stress revealed a significant decrease in key metabolites associated with fats, proteins, and carbohydrates, demonstrating its utility in profiling metabolic responses to environmental stress [23]. Multi-omics integration analysis has been applied to analyze stress responses in fish such as Japanese pufferfish [24] and Nile tilapia [25].

In this study, we assessed the hypoxia tolerance of BTB through controlled hypoxic experiments in a laboratory to confirm the improvement in hypoxia tolerance in the hybrid fish. Subsequently, we analyzed the gill morphological changes, and observed the changes of hemocytes after hypoxic treatment. Additionally, association analysis of transcriptomics and metabolomics revealed the hypoxia tolerance mechanisms of BTB from both gene expression and metabolite regulation aspects. A series of candidate genes related to hypoxic response were identified. This study established a molecular foundation for understanding the mechanisms of hypoxia tolerance in fish.

## 2. Materials and Methods

### 2.1. Experimental Fish and Feeding Protocol

BTB were obtained from Hunan Fish Genetics and Breeding Center, Hunan Normal University, China. All experiments in this study were approved by the Bioethics Committee of the Freshwater Fisheries Research Center (FFRC) of the Chinese Academy of Fisheries Sciences (CAFS) and were conducted in strict accordance with the recommendations in the Guidelines for the Care and Use of Laboratory Animals in China. The fish experiments were approved by the Animal Care Committee of Hunan Normal University (Approval Code: No. 2024-825). Six-month-old experimental fish were collected from an outdoor culture pond. And then experimental fish were transported to the laboratory and raised in 80 L water-recirculating tanks for 15 days to acclimatize laboratory conditions. During this period, the water DO, pH and temperature were 7.5 ± 0.5 mg/L, 7.6 ± 0.2, and 25 ± 1 °C, respectively. DO and temperature were monitored using a dissolved oxygen meter (LDOTM HQ20, Hach Company, Loveland, CO, USA), and pH was measured using a pH meter (PHM 210 standard, Mettler Toledo, Shanghai, China). The fish were fed commercial feed twice a day, with feces removed promptly. Throughout the experiment, fish were maintained on a standard commercial feed for freshwater species and showed no signs of disease, with growth performance within expected parameters.

### 2.2. Hypoxia Treatment

Before hypoxia treatment, the fish were fasted for 24 h. BTB with similar body size and good condition were selected for the experiment. Selected BTB were equally divided into a hypoxia treatment groups and a normoxia control groups, with 10 fish in each group. During the hypoxia experiment period, DO meter (LDOTM HQ20) (LDO101 probe, range 0.1–20.0 mg/L) was used to measure the dissolved oxygen concentration in the water in real-time, and DO in the water was adjusted by filling with nitrogen and pumping oxygen [7]. In the normoxic group, the DO concentration was maintained at 7.5 ± 0.5 mg/L. For the hypoxic group, nitrogen was introduced to adjust the DO concentration from normoxia to 6 mg/L, which was maintained for 1 h. It was then reduced to 3 mg/L for 30 min, followed by a decrease of 0.5 mg/L every 30 min, with each level maintained for 30 min. The DO and time to loss of equilibrium were recorded during the hypoxic treatment period, and the hypoxic treatment was repeated three times. The value of LOE_crit_ (critical oxygen tension for loss of equilibrium) for BTB was calculated using Brett’s equation [26]. LOE_crit_ value represented the DO content in the water when the experimental fish experienced loss of equilibrium.

### 2.3. Sample Collection and Processing

Six experimental fish that exhibited loss of equilibrium were sampled from the hypoxic group, while six fish from the normoxic group were randomly selected as the controls. The experimental fish were anesthetized using 0.2 g/L tricaine methanesulphonate (MS-222) [27]. The gill from the left side of each fish was sampled, which was cut into small pieces along the gill filaments and divided into two parts. One part was transferred into RNase-free tubes and temporarily frozen in liquid nitrogen, and then stored in a −80 °C refrigerator for subsequent RNA extraction, transcriptome sequencing, and qRT-PCR analyses. The other part was immersed in Bouin’s solution and fixed for 24 h, which was used for the subsequent preparation of paraffin sections of gill tissue.

Three fish were randomly selected from each group. For blood collection, fish were rapidly anesthetized in dechlorinated tap water with a concentration of 200 mg/L tricaine methanesulfonate (Sigma, St Louis, MO, USA). Injection syringe was pre-rinsed with heparin, and 200 μL of blood was collected in EDTA-k_2_ an ticoagulation tubes for the detection of blood physiological indices, including red blood cell (RBC) count, white blood cell (WBC) count, hemoglobin (Hb) levels, and hematocrit (HCT).

### 2.4. Gill Histology and Data Analysis

Gill tissues were fixed in Bouin’s solution for 24 h, then transferred to 70% ethanol and subsequently dehydrated in graded ethanol. After being rendered transparent with xylene, samples were embedded in paraffin wax (KH-BL, Kohai Medical, Kohai Medical Technology (Tianjin) Co., Ltd., Tianjin, China). Then, samples were cut into continuous slices with a thickness of 5 μm using a slicer (KHQ330, Kohai Medical). The slices were stained with Hematoxylin-eosin (HE) (G1120, Beijing Solarbio Science & Technology Co., Ltd., Beijing, China), and sealed with neutral gum. Finally, microstructure observation and micrographs of gill were conducted using a microscope (DM500, Leica Microsystems (Shanghai) Trading Co., Ltd., Shanghai, China).

Using ImageJ software (ImageJ2) [28], the gill filaments of each samples were measured, and the length, thickness, spacing, and the height of ILCM of gill lamellae were recorded [29]. All data were analyzed using *t*-test with GraphPad to observe the gill morphological changes after hypoxia treatment.

### 2.5. Blood Sample Analysis

Blood physiological indexes, including RBC, WBC, Hb, and HCT, were measured using a Mindray Veterinary Fully Automatic Hematology Analyzer (BC-2800 vet, Shenzhen Mindray Bio-Medical Electronics Co., Ltd., Shenzhen, China). The data processing method was consistent with that described in Section 2.4.

### 2.6. Transcriptome Sequencing

Total RNA, from gill tissues of 6 samples per group, were extracted using Trizol reagent (Invitrogen, Thermo Fisher Scientific, Shanghai, China), and then purified with the RNeasy Micro kit (Qiagen, Hilden, Germany). The quality of the total RNA was assessed by using a NanoDrop 2000 spectrophotometer (Thermo Fisher Scientific, Shanghai, China) for concentration and purity detection, an Agilent 2100 device (Agilent Technologies, Shanghai, China) for RIN values measurement, and a 1.5% agarose gel electrophoresis for RNA integrity analysis. mRNA was screened and enriched using magnetic beads with Oligo (dT), then cDNA was synthesized and subsequently purified. After purification, the end of cDNA was repaired, and then poly A was added to the 3′ end of cDNA. After adding adapter to the end of cDNA, samples were suffered fragment size selection and PCR amplification. Then, cDNA libraries were constructed. Finally, samples were sequenced using the Illumina Novaseq 6000 sequencing platform (Illumina, Inc., Shanghai, China) [30].

### 2.7. Extraction and Identification of Metabolites

Six samples of each group were used for metabolites extraction. For each sample, 50 mg gill tissue was firstly transferred to 2 mL centrifuge tubes with 6 mm-diameter grinding bead. Extraction solution [methanol/acetonitrile (*v*/*v*) = 4:1] containing 0.02 mg/mL of internal standard (L-2-chlorophenylalanine) was added. Tissues were ground by the frozen tissue grinder (Wonbio-96c, Shanghai wanbo biotechnology Co., Ltd., Shanghai, China) for 6 min (−10 °C, 50 Hz). The samples were ultrasonically processed at low temperature for 30 min (5 °C, 40 kHz), followed by incubation at −20 °C for 30 min. Subsequently, the samples were centrifuged for 15 min (4 °C, 13,000× *g*), and the supernatant was transferred to the injection vial for LC-MS/MS analysis. Quality control (QC) samples were prepared by mixing equal volumes of the supernatants. Liquid chromatography-tandem mass spectrometry (LC-MS/MS) analysis was performed using a Vanquish UHPLC system (Thermo Fisher Scientific, Shanghai, China) [31].

### 2.8. Association Analysis of Metabolome and Transcriptome

Transcriptome and metabolome were combined to determine regulatory pathways involving differentially expressed genes (DEGs) and differential metabolites (DMs) after hypoxia treatment in BTB [24,32,33]. The molecular interaction networks were interpreted through systemically analyzing the relationship between DEGs and DMs. The O2PLS model was constructed to obtain the correlation of DEGs and DMs. The significant KEGG signaling pathways enriched with DEGs and DMs in BTB after hypoxia treatment were annotated using hypergeometric distribution algorithm to reveal the mechanisms underlying hypoxia tolerance in terms of gene expression and metabolic regulation.

### 2.9. Validation of Candidate Genes Using qRT-PCR

Quantitative RT-PCR (qRT-PCR) was applied to confirm the expression of candidate genes identified from transcriptome. Firstly, total RNA was extracted from the gill tissues of both hypoxia-treated and control groups of BTB. cDNA was synthesized using the PrimeScript RT Master Mix kit (Takara Bio (Dalian) Co., Ltd., Dalian, China), and primers were designed using Primer3web version 4.1.0 [34]. Primers were listed in Table S1 of the Supplementary File. qRT-PCR was conducted using the ABI QuantStudio 5 (ABI, Foster City, CA, USA) with the SYBR^®^ Premix Ex Taq kit (Takara). The expression of *egln3*, *im_7150988*, *znf395a*, *hif-1an*, *mknk2b*, *pck2*, *ero1a*, *igfbp-1a*, *vhl*, *bpifcl*, *egln1a*, and *ccna1* from gill tissues was analyzed to verify the reliability of the transcriptome sequencing data, using β-actin as the reference gene. For each group, three biological replicates were conducted. Relative expression levels were calculated using the 2^−ΔΔCt^ method [35].

### 2.10. Statistical Analysis

The non-parametric Kruskal–Wallis test was employed for overall group comparisons. Where significant differences were detected, pairwise comparisons were further assessed using Dunn’s post hoc test. Data analysis was carried out using GraphPad Prism software (Prism 9.x). A limitation is the small sample size, implied by the use of only six specimens for the subsequent omics analyses. A low *n*-value increases the risk of Type II errors and reduces the statistical power and generalizability of the findings.

## 3. Results

### 3.1. Verification of Hypoxic Tolerance Ability of BTB

Laboratorial experiments on hypoxia tolerance of BTB were conducted to confirm the value of critical oxygen tension for loss of equilibrium (LOE_crit_). The experimental results indicated that the average of LOE_crit_ value was 1.0 mg/L for BTB. However, with the same experimental treatment the average of LOE_crit_ value of BSB was 1.2 mg/L [36]. Therefore, hypoxia tolerance of the hybrid fish BTB was increased by 20% compared to its parent BSB.

### 3.2. Gill Morphological Change Under Hypoxic Conditions

**Figure 1:**
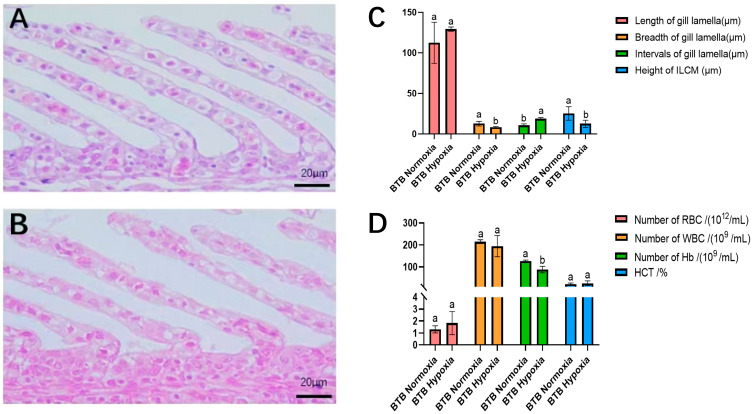
The effects of hypoxic treatment on gill tissue and hematological physiological indices in a hybrid bream (BTB). (**A**) Microstructure of Gill from normoxic group of BTB. (**B**) Microstructure of Gill from hypoxic group of BTB. (**C**) Values of variables on gill lamella in control and hypoxic groups of BTB. (**D**) Blood physiological indicators in control and hypoxic treatment groups of BTB. Data were presented as means ± SD. Bars with different letters indicated statistically different (*p* < 0.05).

Histology of gill tissues revealed that the gill morphology was significantly changed in BTB under hypoxic stress. The statistics of structural indicators of the gill lamellae were analyzed in both normoxic and hypoxic groups. In the normoxic group, the complete and symmetrical distribution of gill structures in BTB indicated that the gills were physiologically and functionally normal (Figure 1A). However, after hypoxia treatment, gill lamellae of the BTB became curved and enlarged at the ends with some epithelial cells swelling or even rupturing, resulting in abnormal physiological morphology (Figure 1B). In addition, the length of gill lamellae became longer, with the diameter of gill lamellae and the height of ILCM significantly decreased (*p* < 0.05), and the lamellae spacing significantly increased in BTB after hypoxia treatment (*p* < 0.05) (Figure 1C). These results revealed that the gill tissues of BTB underwent significant adaptive structural remodeling in response to acute hypoxia exposure.

### 3.3. Blood Analysis

After hypoxia treatment, the number of RBCs increased, while the count of WBCs decreased, and the concentration of Hb was significantly reduced (*p* < 0.05). Besides, the level of HCT was increased under hypoxic conditions (Figure 1D).

### 3.4. Transcriptome Sequencing, DEGs Identification, and Functional Enrichment

To systematically analyze the changes in gene expression under hypoxic stress, 6 samples from the BTB were sequenced with three biological replicates for each group. Using the Illumina HiSeq 6000 platform (Illumina, Inc., Shanghai, China), a total of 43.32 Gb of raw data were generated. After filtering out low-quality reads, 42.53 Gb (98.18%) of sequencing data were obtained. The post-QC data underwent comprehensive statistical and quality evaluations, revealing that the Q20 values of the sequencing data spanned from 98.43% to 98.66%, while the Q30 values ranged between 95.51% and 95.9%. Additionally, the GC content was observed to vary from 45.32% to 48.01%. The high quality of sequencing data ensured the accuracy of subsequent transcriptome analysis.

**Figure 2:**
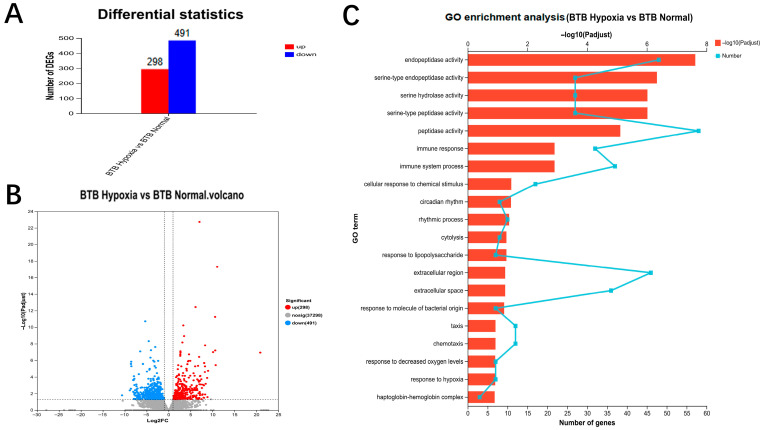
Identification of DEGs in a hybrid bream (BTB) after hypoxia treatment. (**A**) Numbers of identified up- and down-regulated DEGs in BTB, with red representing upregulated genes and blue representing downregulated genes. (**B**) The volcano plot showed significant DEGs in BTB after hypoxia treatment, with red and blue dots indicating upregulated and downregulated DEGs, and grey dots indicating no significant DEGs. (**C**) GO enrichment analysis of differentially expressed genes in BTB.

Using transcriptome data, DEGs were identified with the threshold of *p*-value less than 0.05 and |log2fc| more than 1. A total of 789 DEGs were found between the normoxic and hypoxic groups of BTB. Among these, 298 genes were up-regulated, and 491 genes were down-regulated in the hypoxic group compared to the normoxic group (Figure 2A). These results indicated that hundreds of genes responding to acute hypoxia exhibited different expression in the gill tissues of BTB. Volcano plots were plotted to present DEGs to intuitively demonstrate the changes in gene expression of gill tissues (Figure 2B). Finally, we identified a series of hypoxia-related candidate genes, including *egln3*, *im_7150988*, *znf395a*, *hif-1an*, *mknk2b*, *pck2*, *ero1a*, *igfbp-1a*, *vhl*, *bpifcl*, *egln1a*, and *ccna1*, were significantly differentially expressed in the gill tissue of BTB.

GO annotation analysis was performed on these DEGs identified in BTB, and it found that these DEGs were enriched in 387 GO terms. Among the top 20 terms, in the category of biological processes, DEGs were mainly enriched in “Immune response” (GO:0006955), “Immune system process” (GO:0002376), “Cellular response to chemical stimulus” (GO:0070887), “Response to decreased oxygen levels” (GO: 0036293) and “Response to hypoxia “(GO:0001666). In the category of molecular function, the mainly enriched terms included “Endopeptidase activity” (GO:0004175), and “Peptidase activity” (GO:0008233). Additionally, some DEGs were mainly enriched in two terms of cellular components including “Extracellular region” (GO:0005576) and “Haptoglobin-hemoglobin complex “(GO: 0031838) (Figure 2C). These results implied that genes related to immunity were involved during hypoxic stress.

To understand pathways related to hypoxia, DEGs identified in BTB were mapped to reference pathways in the KEGG database. The KEGG annotation results showed that the top 20 KEGG pathways included Apoptosis, the Natural killer cell mediated cytotoxicity, the MAPK signaling pathway, the TNF signaling pathway, the Viral protein interaction with cytokine and cytokine receptor, the Toll-like receptor signaling pathway (Figure S1). The KEGG annotation results also showed that DEGs were enriched in the hypoxia-related pathways including HIF-1 signaling pathway and FoxO signaling pathway. The KEGG annotation results indicated, to response acute hypoxic, many pathways were involved such as immune-related pathways, but not just hypoxia-related pathways.

### 3.5. Metabolomic Analysis

Metabolomics analysis was conducted in BTB to identify metabolites involved in hypoxia response. A total of 365 metabolites were identified in the positive mode and 458 metabolites in the negative mode. Partial Least Squares Discriminant Analysis (PLS-DA) was performed to distinguish the differences in metabolites between normoxic and hypoxic groups. This analysis showed that the metabolic spectrums were distinctly separated into different groups in both positive and negative modes (Figure S2), which contributed to searching DMs related to acute hypoxia response. To avoid overfitting of the PLS-DA model, the model was verified using an alternative test based on 200 tests. The R^2^Y metric, which describes the percentage of variation explained by the model, was consistently greater than the Q^2^ metric, which describes the predictive ability of the model. Both Q^2^ and R^2^Y were close to 1 across all modes, indicating that the model exhibited good stability and was not prone to overfitting. Therefore, the model was reliable and effective for identifying DMs in response to acute hypoxic stress in BTB.

The threshold of variable importance in the projection (VIP) value more than 1 and adjusted *p*-value less than 0.05 was used to screen DMs from BTB. A total of 108 DMs were identified in BTB, among which 78 DMs were up-regulated and 30 were down-regulated by comparing hypoxic group to normoxic group. The significance of DMs between the hypoxia-treated group and the normoxic group was visually presented in volcano plot, indicating the DMs closely related to the metabolic adaptation mechanisms under hypoxic stress (Figure S3). The top 10 DMs are listed in Table 1.

**Figure 3:**
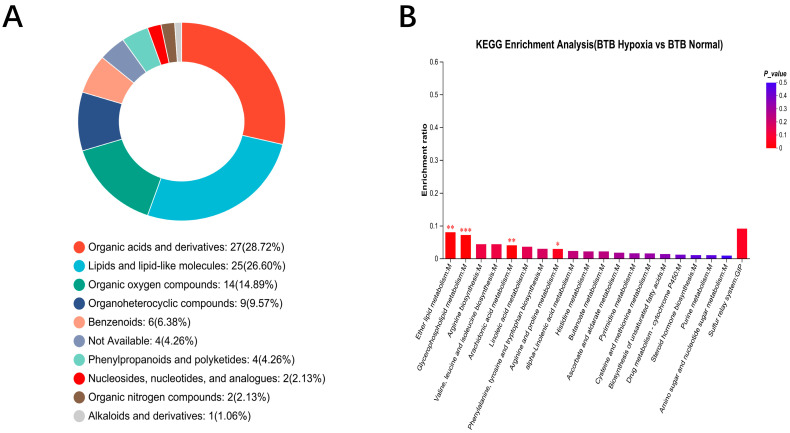
Classification analysis of HMDB compounds and KEGG enrichment analysis of DMs in a hybrid bream (BTB) after hypoxia treatment. (**A**) DMs were classified into 10 HMDB superclasses. (**B**) KEGG enrichment analysis of DMs in BTB. The asterisk (*) indicated a significant KEGG pathway (*p* < 0.05). The asterisk (**) indicated a significant KEGG pathway (*p* < 0.01). The asterisk (***) indicated a significant KEGG pathway (*p* < 0.001).

Classification results based on the Human Metabolome Database (HMDB) revealed that DMs after hypoxia-tolerant treatment were enriched in 10 superclasses, with predominant annotations in Organic acids and derivatives, Lipids and lipid-like molecules and Organic oxygen compounds (Figure 3A). KEGG pathway enrichment analysis was performed. DMs were enriched in 23 metabolic pathways, including significantly enriched in Arginine and proline metabolism, Ether lipid metabolism, Arachidonic acid metabolism, and Glycerophospholipid metabolism (Figure 3B). The DMs of BTB were primarily enriched in lipid metabolism and amino acid metabolism pathways.

### 3.6. Association Analysis of Transcriptome and Metabolome

**Figure 4:**
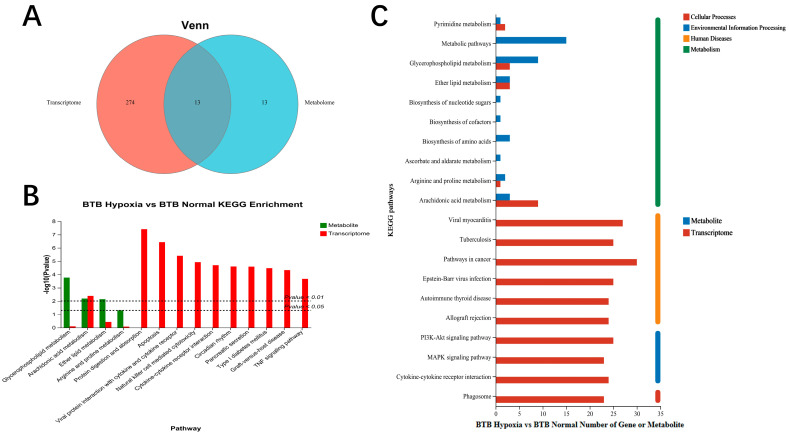
KEGG pathway annotation analysis and KEGG pathway enrichment analysis of DMs and DEGs between a hybrid bream (BTB) after hypoxia treatment. (**A**) Venn plot showed common and specific KEGG pathway of DEGs and DMs in BTB. (**B**) KEGG pathway enrichment analysis of DMs and DEGs in BTB. (**C**) KEGG pathway annotation analysis of DMs and DEGs in BTB.

To identify the pathways involving both DMs and DEGs in the BTB, the DMs and DEGs from the set (BTB Hypoxia vs. BTB Normal) were mapped to KEGG pathways. The Venn diagram showed that DMs and DEGs are co-enriched in 13 KEGG pathways (Figure 4A). The results revealed that pathways including Glycerophospholipid metabolism, Ether lipid metabolism, Arachidonic acid metabolism, Arginine and proline metabolism were enriched in BTB (Figure 4C). Meanwhile, the KEGG enrichment analysis revealed that both DMs and DEGs were significantly co-enriched in Arachidonic acid metabolism (Figure 4B). The integrated analysis of DMs and DEGs in iPath 3.0 revealed that under hypoxic stress, energy metabolism and lipid metabolism pathways in BTB were altered (Figure S4). These results indicated that hypoxic stress triggered the expression of genes and metabolites associated with lipid and amino acid metabolism pathways, while simultaneously modulating energy metabolism and lipid metabolism to facilitate hypoxia adaptation in BTB.

### 3.7. Validation of Candidate Genes Using qRT-PCR

**Figure 5:**
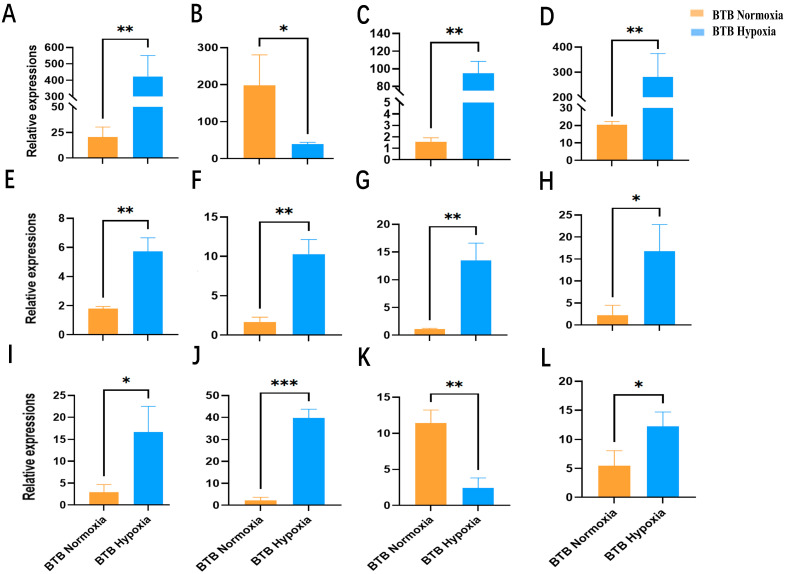
QPCR validation of 12 significant DEGs identified from BTB after hypoxia treatment. (**A**) Egl-9 family hypoxia inducible factor 3 (*egln3*); (**B**) Golgi-associated plant pathogenesis-related protein 1 (*im_7150988*); (**C**) Zinc finger protein 395a (*znf395a*); (**D**) Hypoxia inducible factor 1 subunit alpha inhibitor (*hif-1an*); (**E**) MAPK interacting serine/threonine kinase 2b (*mknk2b*); (**F**) Phosphoenolpyruvate carboxykinase 2 (mitochondrial) (*pck2*); (**G**) Endoplasmic reticulum oxidoreductase 1 alpha (*ero1a*); (**H**) Insulin-like growth factor binding protein 1a, transcript variant X1 (*igfbp-1a*); (**I**) von Hippel-Lindau tumor suppressor (*vhl*); (**J**) Egl-9 family hypoxia-inducible factor 1a (*egln1a*); (**K**) BPI fold containing family C, like, transcript variant X2 (*bpifcl*); (**L**) Cyclin A1 (*ccna1*). The asterisk (*) indicated a significant difference between the control group (normoxic group) and the treatment group (hypoxic group) (*p* < 0.05). The asterisk (**) indicated a significant difference (*p* < 0.01) between the control group and the treatment group. The asterisk (***) indicated a significant difference (*p* < 0.001) between the control group and the treatment group, while ns indicated no significant difference between the two groups.

To validate the quality of RNA-seq data, twelve candidate genes involved in the hypoxia response were selected from transcriptome analysis after hypoxia treatment. These genes include Egl-9 family hypoxia inducible factor 3 (*egln3*); Golgi-associated plant pathogenesis-related protein 1 (*im_7150988*); Zinc finger protein 395a (*znf395a*); Hypoxia inducible factor 1 subunit alpha inhibitor (*hif-1an*); MAPK interacting serine/threonine kinase 2b (*mknk2b*); Phosphoenolpyruvate carboxykinase 2 (mitochondrial) (*pck2*); Endoplasmic reticulum oxidoreductase 1 alpha (*ero1a*); Insulin-like growth factor binding protein 1a, transcript variant X1 (*igfbp-1a*); von Hippel–Lindau tumor suppressor (*vhl*); Egl-9 family hypoxia-inducible factor 1a (*egln1a*); BPI fold containing family C, like, transcript variant X2 (*bpifcl*); Cyclin A1 (*ccna1*). QPCR was performed with three biological replicates and three technical replicates. The qPCR results showed that the gene expression levels of *egln3*, *znf395a*, *hif-1an*, *mknk2b*, *pck2*, *ero1a*, *igfbp-1a*, *vhl*, *egln1a*, and *ccna1* were significantly up-regulated, while *im_7150988* and *bpifcl* were significantly down-regulated in BTB after hypoxic treatment (Figure 5). The expression patterns of these twelve genes were consistent with the transcriptome analysis results, further validating the reliability of the transcriptome sequencing data.

## 4. Discussion

BSB is an economic freshwater fish with sensitive to hypoxia which largely constrained its aquaculture products. BTB is generated by hybridizing and backcrossing between BSB and TC [36]. BTB exhibits significantly enhanced hypoxia tolerance. It was observed during aquaculture practice with a significantly lower frequency of head floating to the surface of the water body compared to its parent BSB. In this study, laboratory experiments demonstrated that BTB had a lower LOE_crit_ value of 1.0 mg/L, compared to 1.2 mg/L for its parent BSB [36]. Previous studies showed that important economic traits could be improved through hybridization [16]. Our study demonstrated that the enhanced hypoxia tolerance of BTB, a hybrid generated from multiple crosses of BSB and TC, results from hybridization. This confirmed hybridization as an effective strategy for developing superior germplasm with traits like improved hypoxia tolerance and rapid growth for aquaculture.

The gill is a primary respiratory organ in fish and plays key roles in osmoregulation, metabolite excretion, and immune regulation [37]. Research indicated that fish adapt to hypoxic environments by modifying the structure of their gills [38]. In addition, apoptosis of interlamellar tissue and elongation of gill filament were observed in crucian carp when exposed to hypoxic conditions [39]. Consistent with previous studies, our results demonstrated that BTB enhanced its hypoxia tolerance through gill remodeling: the elongation of lamellae, a marked reduction in their diameter, a substantial increase in lamellar spacing, and a significant decrease in ILCM height collectively increased the respiratory surface area and improved water contact efficiency.

Red blood cells (RBCs) play a central role in oxygen transport, and hypoxia-tolerant species often enhance oxygen-carrying capacity through RBC proliferation. For instance, under hypoxic stress, turbot (*Scophthalmus maximus*) elevates RBC production to improve oxygen delivery [40]. Under hypoxic conditions, juvenile turbot exhibited upregulated red blood cell (RBC) counts, hemoglobin (Hb) levels, and hematocrit (HCT) [41]. Similar changes of RBC counts and HCT were also observed in BTB under hypoxia. However, in contrast to these studies, BTB displayed a significant reduction in Hb concentration (*p* < 0.05), suggesting a unique adaptive strategy to optimize oxygen transport efficiency. This divergence may reflect species-specific regulation of Hb thresholds, as blood oxygen-carrying capacity is critically dependent on Hb concentration [42]. By reducing Hb to an optimal level, BTB could enhance oxygen utilization while maintaining physiological flexibility under declining dissolved oxygen. Notably, white blood cell (WBC) counts showed nonsignificant fluctuations post-hypoxia, potentially indicating prioritized resource allocation toward oxygen transport over transient immune responses during acute stress.

Comparing to physiological tolerance and gill remodeling, the multi-omics approach revealed that hypoxia adaptation in BTB involves specific alterations in lipid and amino acid metabolism pathways and coordinated changes in key genes and metabolites, providing a systemic molecular perspective. The hypoxia-inducible factor 1 (HIF-1) signaling pathway is evolutionarily conserved and central to cellular hypoxia adaptation, with HIF-1α acting as a master transcriptional regulator [43,44]. In teleosts, hypoxia triggers *hif-1a* upregulation to enhance oxygen delivery and reduce metabolic demand [45]. Downstream genes of *hif-1* have been reported to be involved in oxygen delivery and the reduction in oxygen consumption [46]. In BTB, hypoxic stress induced pronounced upregulation of HIF-1 pathway genes (*egln1a*, *egln3*, *vhl*, *mknk2b*), suggesting robust activation of oxygen-sensing mechanisms. The FoxO signaling pathway plays a significant role in the hypoxia response, influencing processes such as oxidative stress resistance, immune regulation, apoptosis, autophagy regulation, glycolysis, and gluconeogenesis [47,48]. In this study, *pck2* (phosphoenolpyruvate carboxykinase 2) was associated with the FoxO signaling pathway, which was upregulated in BTB after hypoxic treatment. The PCK2 is the mitochondrial phosphoenolpyruvate carboxykinase [49], and promotes gluconeogenesis to reduce oxygen consumption, thereby maintaining intracellular energy balance under hypoxic stress [12]. Insulin-like growth factor binding protein 1 (IGFBP1) downregulates the activity of insulin-like growth factors (IGFs), modulating cell growth and development under hypoxia stress [50]. Gene *igfbp-1a* was significantly upregulated in BTB after hypoxia treatment. Consistent with this result, hypoxia induction upregulated the expression of *igfbp1* in zebrafish (*Danio rerio*) embryos [51]. Overexpression of *igfbp1* resulted in slow growth of zebrafish embryos under normoxic conditions [52]. The association analysis showed Arachidonic acid and Glycerophospholipid metabolism were co-enriched in metabolomics and transcriptomics, suggesting a plausible model where hypoxia-induced membrane remodeling and inflammatory signaling are functionally linked. Arachidonic acid, a key lipid messenger, could be the nexus, released from remodeled phospholipids to activate signaling pathways that orchestrate the observed immune and metabolic responses. Collectively, these findings revealed that BTB coordinated HIF-1 and FoxO pathways to optimize oxygen utilization and metabolic efficiency, while modulating IGF signaling to balance energy allocation—a strategic adaptation critical for survival in hypoxic aquaculture environments.

Transcriptome sequencing revealed that DEGs were enriched in immune-related pathways in BTB, suggesting an activation of the immune system under hypoxic stress. In large yellow croaker, transcriptome analysis suggested an early adjustment pattern of fish immune response to cope with hypoxia stress [53]. In addition, chronic hypoxia modulated the expression of important immune-related genes putatively altering the immune response [54]. The MAPK signaling pathway participates in regulating and activating immune responses, which is a key intracellular signaling cascade that regulates stress response [55,56]. In BTB, we identified a series of upregulated DEGs involved in the MAPK signaling pathway such as *mknk2b*, *efna1b*, and cytoplasmic phospholipase A2 zeta-like (*LOC125266034*). This implied that immune and hypoxia responses are co-activated through a shared signaling pathway under hypoxic stress in fish. These findings indicated that the immune system was activated under hypoxic stress and contributed to hypoxia adaptation.

While this study provides a multi-omics perspective on the hypoxic response in BTB, several limitations should be acknowledged. The investigation focused exclusively on the effects of acute hypoxia and was restricted to gill tissues. Although gills are the primary site of oxygen exchange, examining other crucial organs like the liver and brain would offer a more comprehensive understanding of the systemic adaptation mechanisms. A key difference between this study and previous physiological investigations is the integration of transcriptomic and metabolomic data, which moves beyond descriptive tolerance metrics and gill remodeling to reveal the underlying molecular network. This approach uniquely identified that hypoxia adaptation involves a coordinated rewiring of lipid and amino acid metabolism pathways (e.g., Arachidonic acid and Glycerophospholipid metabolism), coupled with immune-inflammatory signaling. Future work should focus on chronic hypoxia exposure and multi-organ analysis to build a systems-level model. The proposed mechanistic link between membrane phospholipid metabolism and inflammatory signaling requires functional validation, for instance, through the pharmacological inhibition of key enzymes like phospholipase A2 to confirm its role in the hypoxic response.

## 5. Conclusions

In this study, we investigated the mechanisms of hypoxia tolerance in BTB by combining histology, transcriptomics and metabolomics. The hypoxia tolerance experiment showed that LOE_crit_ value of BTB was relative lower than its parent BSB, indicating the ability of hypoxia tolerant of BTB was improved through hybridization. The histological results of the gills revealed structure modifications that facilitate adaptation to hypoxic stress. Blood analysis revealed that the blood physiological indicators of the species also underwent changes to adapt to hypoxic conditions. Association analysis of transcriptomics and metabolomics had revealed pathways associated with hypoxia tolerance. Additionally, twelve DEGs related to hypoxia response were screened and identified. These findings indicated potential mechanisms for hypoxia tolerance in hybrid fish, and provided genetic basis for generating germplasm resources with improved stress resistance for aquaculture.

## Figures and Tables

**Table 1:** Top 10 DMs in a hybrid bream (BTB) after hypoxia treatment.

Name	Class	Adjusted *p*-Value	VIP	FC	Regulate
Argininic acid	Carboxylic acids and derivatives	0.0005	3.26	1.229	up
S-Adenosylmethionine	S-Adenosylmethionine	0.008	3.09	1.282	up
2-Hydroxydecanedioic acid	Hydroxy acids and derivatives	0.001	3.05	0.82	down
Lysylthreonine	Carboxylic acids and derivatives	0.007	2.85	1.22	up
Nopalinic acid	Carboxylic acids and derivatives	0.009	2.75	1.202	up
Glycerylphosphorylcholine	Glycerophospholipids	0.004	2.67	1.126	up
Meclizine	Benzene and substituted derivatives	0.004	2.60	1.093	up
APGPR Enterostatin	Benzene and substituted derivatives	0.007	2.04	1.105	up
Diphenyl disulfide	Benzene and substituted derivatives	0.006	2.03	1.082	up
N-a-Acetyl-L-arginine	Carboxylic acids and derivatives	0.006	1.87	1.065	up

## Data Availability

The data presented in this study are available on request from the corresponding author.

## Supplementary Materials

The following supporting information can be downloaded at: https://www.mdpi.com/article/10.3390/biology14101462/s1, Table S1: Primer sequences for qPCR validation; Figure S1: KEGG analysis of differentially expressed genes; Figure S2: Partial least squares discriminant analysis (PLS-DA) for detecting gill metabolites in LC-MS data; Figure S3: Identification of differential metabolites in a hybrid bream (BTB) after hypoxia treatment; Figure S4: iPath 3.0 visualization of metabolic pathways integrating transcriptomic and metabolomic data.
