# Supplementary material for: Research on the Mechanism of Hypoxia Tolerance of a Hybrid Fish Using Transcriptomics and Metabolomics
Source: Biology (Basel). 2025 Oct 21;14(10):1462. doi: 10.3390/biology14101462 (PMC12561119; doi:10.3390/biology14101462)
Supplement: Supplementary file 1 [file biology-14-01462-s001.zip › biology-3922748-supplementary.pdf]

Research on the Mechanism of Hypoxia Tolerance of a Hybrid Fish Using Transcriptomics and Metabolomics

Yuhua Tang [1,†], Jiayi Yang [1,†], Chunchun Zhu [1], Hong Zhang [1], Li Hu [1], Wenting Rao [1], Xinxin Yu [1], Ming Wen [1,2,*], Min Tao [1,2] and Shaojun Liu [1,2,*]

[1] Engineering Research Center of Polyploid Fish Reproduction and Breeding of the State Education Ministry, College of Life Sciences, Hunan Normal University, Changsha 410081, China; 19310032129@163.com (Y.T.); 15067848138@163.com (J.Y.); 19307411565@163.com (C.Z.); zhanghong98@hunnu.edu.cn (H.Z.); 17872348328@163.com (L.H.); rdmnei@outlook.com (W.R.); yxx1758155428@163.com (X.Y.); minmindiu@126.com (M.T.)

[2] Yuelushan Laboratory, Changsha 410128, China

* Correspondence: ming.wen@hunnu.edu.cn (M.W.); lsj@hunnu.edu.cn (S.L.)

† These authors contributed equally to this work.

Keywords: hybrid fish; transcriptomics; metabolomics; hypoxia tolerance; gills

Supplementary Table S1 | Primer sequences for qPCR validation.

Supplementary Figure S1 | KEGG analysis of differentially expressed genes.

Supplementary Figure S2 | Partial least squares discriminant analysis (PLS-DA) for detecting gill metabolites in LC-MS data.

Supplementary Figure S3 | Identification of differential metabolites in a hybrid bream (BTB) after hypoxia treatment.

Supplementary Figure S4 | iPath 3.0 visualization of metabolic pathways integrating transcriptomic and metabolomic data.

Table S1: Primer sequences for qPCR validation.

names	Primer Sequence (5' - 3')
egln3	F: CCAGGAAATGGAGCAGGATA<br>R: GGGTTCCTACGATCTGACCA
im_7150988	F: CACAGGGAAGGAGGCTGTAG<br>R: ACGACAAAAACCGTGTTTCC
znf395a	F: ACCCGTTCAGGTTGTGACTC<br>R: ATCCTCCTGGAAGTGCAATG
hif-1an	F: GTGGACACGATGCATCAAAC<br>R: TGGTGTTACATTGCCTTCCA
mknk2b	F: ACATCCCAGATGCCAAAAAG<br>R: CTGTGTCCTGGCCTCTTCTC
pck2	F: CAGGCTGGAAGGTTGAGTGT<br>R: GGCATGAGGGTTGGTCTTTA
ero1a	F: GAGCGTTTCACCGGCTATAA<br>R: CTCCAGCCAGCGATAGAAAC
igfbp-1a	F: GGAGCAGGGTCCTTGTCATA<br>R: CCATCCAGAGACGATTCACA
vhl	F: GCAAGTCTCAATTGCCAACA<br>R: CTGGGTCTCTGAGCAAGGTC
bpifcl	F: ATTCGGCAGCTTATGCATTC<br>R: TGCATCTCCATGTTGGGATA
egln1a	F: AAAGCTCAGTTTGCGGACAT<br>R: CGCTCATCAGCATCGAAATA
ccna1	F: CCTCCGAGGATGTCTTGTGT<br>R: GCCAACTTCAACCAACCAGT

Designed QPCR primers for candidate gene regions resistant to hypoxia

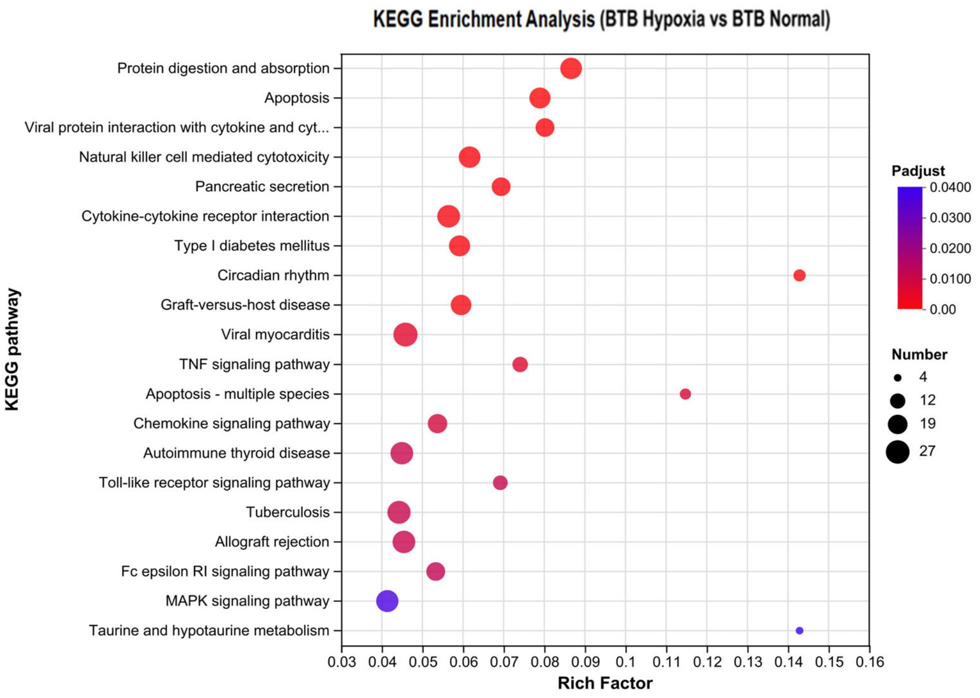

Figure S1: The top 20 KEGG (Kyoto Encyclopedia of Genes and Genomes) enrichment analysis of differentially expressed genes in response to hypoxia. KEGG enrichment analysis of differentially expressed genes in BTB.

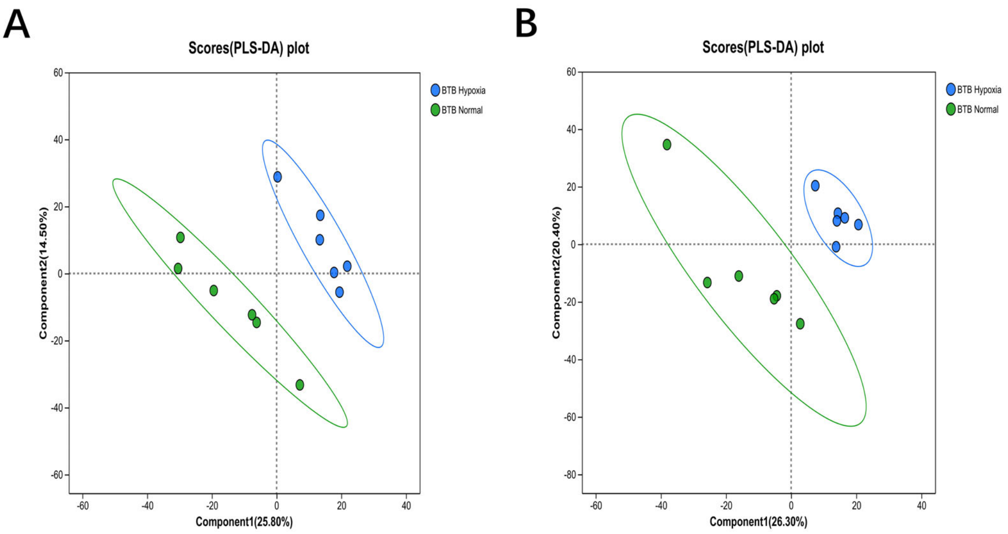

Figure S2: The partial least squares discriminant analysis (PLS-DA) plots of the differential metabolites in LC-MS data in cationic and anionic modes. (A) cationic mode PLS-DA score graph, BTB, R2X =0.468, R2Y=0.997, Q2= 0.734. (B) anionic mode PLS-DA score graph, BTB, R2X =0.619, R2Y=0.978, Q2= 0.78.

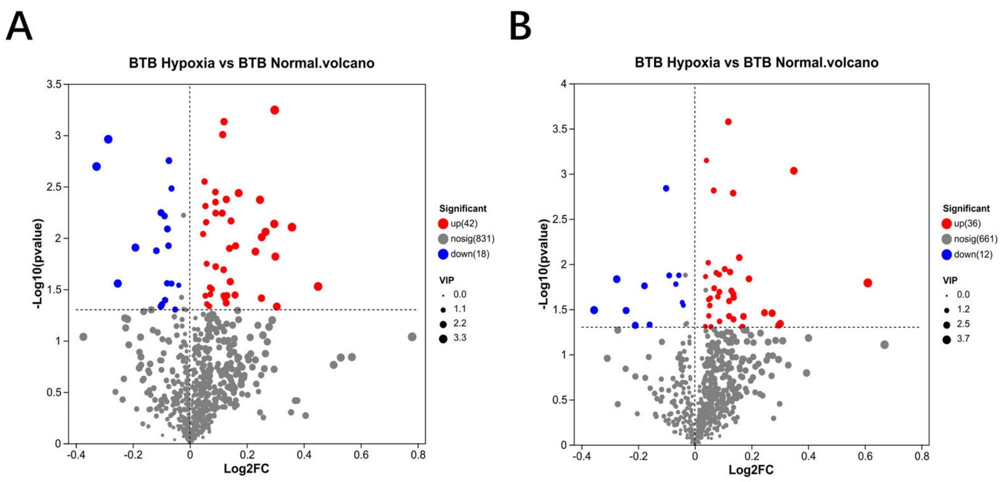

Figure S3: Differential metabolites analysis in a hybrid bream (BTB) after hypoxia treatment. (A) The volcano plot showed significant DMs of cationic mode in BTB after hypoxia treatment. (B) The volcano plot showed significant DMs of anionic mode in BTB after hypoxia treatment, the red and blue dots in the volcano plot represent up-regulated and down-regulated DMs, and grey dots indicated no significant DMs.

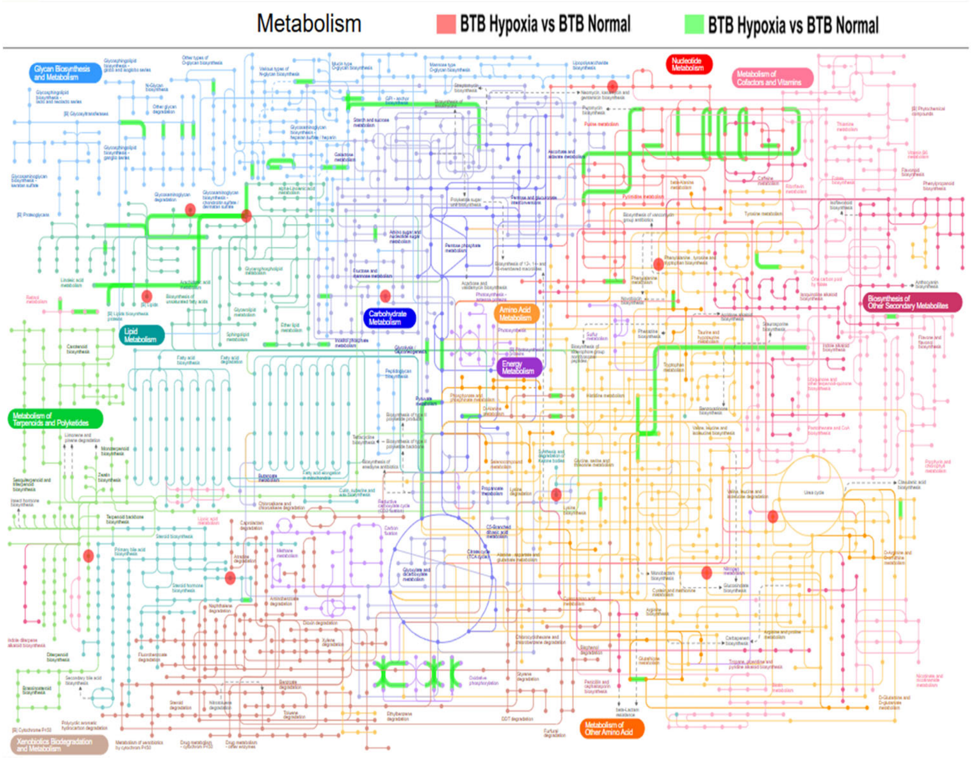

Figure S4: Visualization of metabolic pathways integrating transcriptomic and metabolomic data. The iPath 3.0 diagram represents metabolic pathways annotated by both metabolite and gene sets. Red-colored pathways denote metabolite set annotations, while green-colored pathways indicate gene set annotations.
